# Beyond equity, diversity and inclusion: the power of intersectionality in transforming workforces

**DOI:** 10.1016/j.lana.2025.100991

**Published:** 2025-01-13

**Authors:** The Lancet Regional Health – Americas

Intersectionality, a term coined by Kimberlé Crenshaw in 1989, emphasises the interconnectedness of various aspects of an individual's social identity and how these intersect to shape experiences and opportunities. Rooted in the late 1970s Black feminist thought, intersectionality understands that social categorizations such as race, gender, and class create overlapping and interdependent systems of discrimination. Social identities' impacts are not siloed or independent; rather, they create unique constellations of oppression and privilege. Individuals with intersecting minoritised identities experience a set of oppressions and violence that go beyond the sum of each of their identities. Recognising, understanding and accounting for the unique impact of intersectionality on how people experience various structural systems of oppression in our society should be the starting point for creating an inclusive environment for work, education, services and social interactions.

The diversity, equity, and inclusion (DEI) framework, rooted in the 1960s US Civil Rights Movement, began with efforts to increase the representation of historically disadvantaged groups in the workplace. It has since evolved to include equity and inclusion, to secure not just diverse representation, but also equitable opportunities and an inclusive environment for all individuals. The importance of DEI initiatives is increasingly recognised within all sectors of our society. Medicine and science are areas with deep roots in racist and eugenic practices, with no shortage of examples of systemic violence against marginalised populations. Medical education and the healthcare workforce still reflect historical inequalities, making DEI initiatives crucial for achieving equity and ensuring everyone has access to the care they need, regardless of their background or circumstances.

“Diversity” encompasses a broad spectrum of differences, including race, gender, age, religion, disability, and socioeconomic background. In the context of healthcare and scientific workforces, understanding and addressing these intersecting identities is crucial for effective DEI initiatives. The series “Intersectionality in the Healthcare and Scientific Workforces” published in this month's issue delves into this topic, challenging us to use an intersectionality lens to approach DEI efforts. The three-paper series is set to show how including intersectionality can significantly enhance DEI initiatives by understanding specific structural barriers to access, retention and growth experienced by individuals with intersecting identities.

The series contrasts the US and Latin American perspectives on intersectionality, highlighting the central role of historical and social context in understanding the different systems of structural oppression that continue to prevail in each country and region. Nevertheless, through singular systems, systematic exclusion continues to affect entry and progression in science and medicine careers for all Americans with intersecting minoritised identities, hindering the progress towards a diverse and inclusive workforce that is more empowered and equipped to deliver equitable care.

Diverse healthcare teams are better positioned to understand and address the unique needs of varied patient populations. Patients often feel more comfortable and trust healthcare providers who understand their cultural nuances. This empathic connection leads to improved communication, more accurate diagnoses, and effective treatment plans. On the healthcare worker side, working in a diverse environment fosters creativity, builds self-esteem, broadens perspectives and builds empathy in all team members.

Creating a healthcare workforce that reflects the population it serves is an essential and already overdue challenge. Sadly, despite the progress made, DEI initiatives are currently facing significant threats in the United States. Republican lawmakers in more than 30 states have introduced or passed more than 100 bills to either restrict or regulate DEI initiatives in several industries, including higher education, and President-elect Donald Trump has signalled his intention to dismantle DEI initiatives in his next term. These developments pose a serious risk to the advancements made in promoting DEI within healthcare and education, with a direct impact on the next generation of healthcare workers and providers. The vilification of DEI efforts can create a hostile environment for individuals from diverse backgrounds, discouraging them from pursuing careers in healthcare and science and perpetuating the oppressive *status quo*. The impact of these bans will likely extend beyond US borders, potentially undermining DEI efforts in other countries in the Americas that look to the US as a model for policies or have aligned political views, such as Argentina.

In stark contrast to the current political movements, at *The Lancet Regional Health—Americas* we believe that, rather than limiting DEI initiatives, the fields of science, medicine, and education should expand their efforts to include an ‘intersectionality lens.’ Only by fully understanding the complexity of our people can we create a more just and equitable society, where everyone is enabled to thrive. We encourage readers to engage with the series and apply the intersectionality principles in their work. By continuing the conversation and efforts towards a more inclusive and equitable future, we can ensure that the progress made in DEI is not only preserved but also advanced.

